# Familial atypical parkinsonism with rare variant in *VPS35* and *FBXO7* genes: A case report

**DOI:** 10.1097/MD.0000000000005398

**Published:** 2016-11-18

**Authors:** Tereza Bartonikova, Katerina Mensikova, Lenka Mikulicova, Radek Vodicka, Radek Vrtel, Marek Godava, Miroslav Vastik, Michaela Kaiserova, Pavel Otruba, Iva Dolinova, Martin Nevrly, Petr Kanovsky

**Affiliations:** a Department of Neurology; b Department of Medical Genetics and Fetal Medicine, Faculty of Medicine and Dentistry, Palacky University, University Hospital, Olomouc; c Department of Nanomaterials in Natural Sciences, Technical University, Liberec, Czech Republic.

**Keywords:** atypical parkinsonism, FBXO7, hereditary parkinsonism, VPS35

## Abstract

**Background:**

A higher prevalence of parkinsonism was recently identified in southeastern Moravia (Czech Republic). Further research confirmed 3 large pedigrees with familial autosomal-dominant parkinsonism spanning 5 generations.

**Methods:**

This case report concerns a patient belonging to one of these 3 pedigrees, in whom motor and oculomotor symptoms were accompanied by frontal-type dementia, who finally developed a clinical phenotype of progressive supranuclear palsy. Molecular genetic examinations were performed due to the positive family history.

**Results:**

No previously described causal mutation was found. After filtering against common variants (minor allele frequency (MAF) < 0.01), 2 noncoding and 1 synonymous rare mutation potentially associable with parkinsonism were identified: *GIGYF2—GRB10* Interacting GYF Protein 2, *PARK11* (c.∗2030G > A, rs115669549); *VPS35* gene—vacuolar protein sorting 35, *PARK17* (c.102 + 33G > A, rs192115886); and *FBXO7*—F-box only protein 7 gene, *PARK15* (c.540A > G, rs41311141).

**Conclusion:**

As to the changes in the *FBXO7* and *VPS35* genes (despite phylogenetic conservation in primates), probably neither the *FBXO7* nor the *VPS35* variants will be direct causal mutations. Both described variants, and possibly the influence of their combination, could increase the risk of the disease.

## Abstract 2

Supplemental Digital Content is available in the text

## 1 Introduction

**Figure 1:**
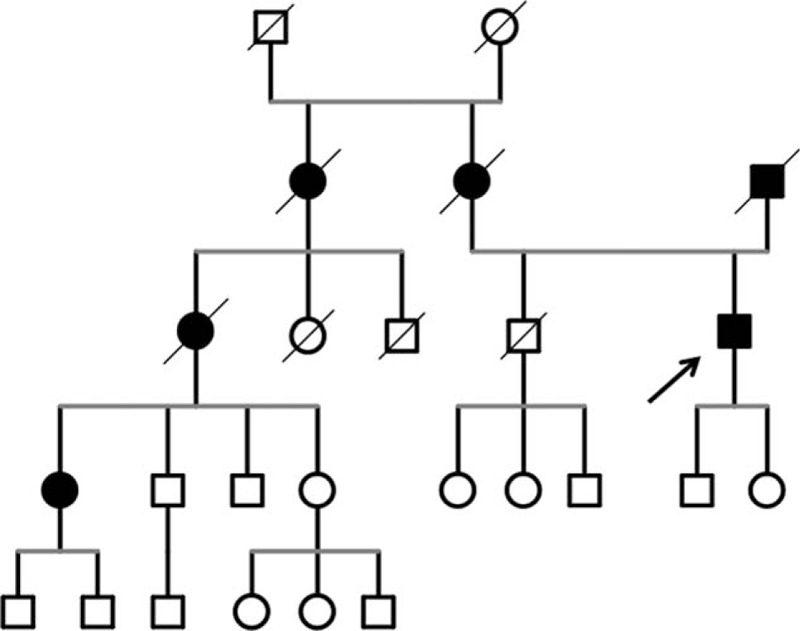
Part of the pedigree in which the familiar atypical parkinsonism has been identified (see Ref. ^[3]^). The patient is indicated by the arrow.

A recent epidemiological study performed in southeastern Moravia uncovered a surprisingly high prevalence of parkinsonism compared to other European countries, with an overall 2.8% prevalence in the population over 50 years of age. Subsequently, 3 large pedigrees with an autosomal-dominant (AD) trait with incomplete penetration were identified, all originating in 1 of the local villages.^[1–3]^ Our patient belongs to 1 of these pedigrees (Fig. 1).

## 2 Case report

The patient is an 82-year-old male. The patient's father had Parkinson disease and died at the age of 74 years; his mother was treated for Parkinson disease with dementia and died of unknown reasons associated with the central nervous system at the age of 57 years in a psychiatric asylum. The patient's paternal grandfather died at a young age in a foresting accident. The paternal grandmother died in middle age due to “nerves”; this information was only reported by the family as the medical records in this area did not start before 1923, when the first general practitioner settled there.

**Figure 2:**
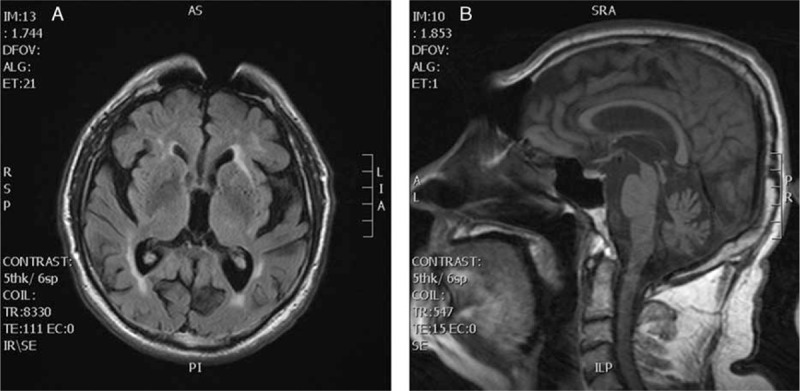
Horizontal FLAIR MRI sequence showing diffuse brain atrophy with postischemic hyperintensities in white matter bilaterally and nonhomogeneous signal and blurred contours of the basal ganglia (A). Sagittal T1-weighted MRI showing regular configuration of the brainstem without noticeable atrophy (B). FLAIR = fluid-attenuated inversion recovery, MRI = magnetic resonance imaging.

The patient previously had experienced only arterial hypertension. The first neurological symptoms appeared at the age of 66 years with slowness of movement, gait initiation failure, and shortened steps; however, he paid no attention to these symptoms until the disease had considerably worsened. He was seen by a movement disorder specialist at the age of 72 years, when general slowness, asymmetric rigidity and bradykinesia, resting tremor of both upper extremities, and gait disorder were present. Levodopa (L-DOPA) treatment was initiated at a daily dose of 800 mg with only a poor response. He was admitted for the first time to the neurological ward at the age of 73 years. Hypomimia and slurred speech were observed, together with bradykinesia, general rigidity, bilateral resting tremor of the hand, positive applauding sign, postural instability, and gait disorder; the cognitive disorder was also more prominent, with an mini mental state examination (MMSE) score of 23/30. Brain magnetic resonance imaging (MRI) revealed brain atrophy, minor postischemic hyperintensities, and blurred basal ganglia contours. Brain MRI revealed diffuse brain atrophy with postischemic hyperintensities in white matter bilaterally. Nonhomogeneous signal and blurred contours of the basal ganglia were present (Fig. 2A). Configuration of the brainstem was regular without noticeable atrophy (Fig. 2B).

At the age of 74 years, there was a rapid progression of bradykinesia and postural instability with falls. At the age of 75 years, visual hallucinations appeared, with the patient frequently reporting “strangers” in his neighborhood. Quetiapine was subsequently added to the medication at a dose of 50 mg/d; the hallucinations disappeared after a few months, and quetiapine was then removed from the medication. Cognitive deficit further deteriorated, and rivastigmine was therefore added.

At the age of 77 years, further bradykinesia and deterioration of postural stability and gait was identified, together with drowsiness and apathy; there was also significant supranuclear gaze palsy. The deterioration in MMSE, with a score of 16/30, confirmed the progressive cognitive deficit. Over the course of the next 3 years, the motor and cognitive deterioration continued due to postural instability, the patient was unable to walk; he experienced generalized bradykinesia and rigidity, together with hypomimia, dysarthria, supranuclear gaze palsy, apraxia of lid opening, and severe dementia. He was completely dependent on home care and family support (see Video [Video shows the patient in his home in autumn 2014. Note the presence of blepharospasm, apraxia of lid opening, dysarthria. At the time of video recording, the patient was unable to stand and walk, so the video was done only with patient sitting.]). Symptoms of autonomic dysfunction were not present throughout the course of the disease.

**Figure 3:**
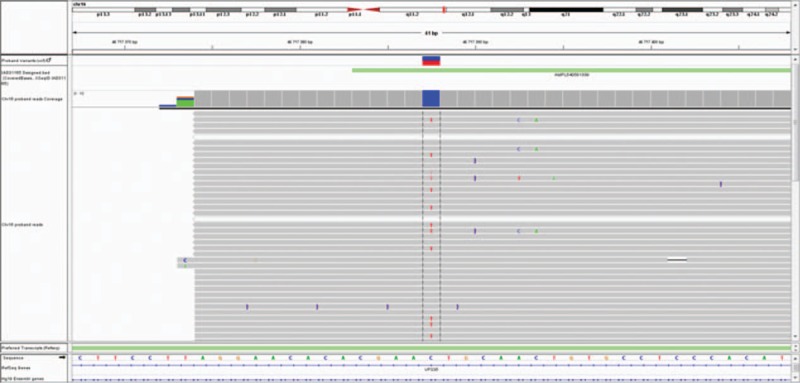
Visualization of vacuolar protein sorting 35 gene variant (NM_018206.4:c.102 + 33G > A, rs192115886) in integrative genomics viewer. The variant T (complementary to the reverse base A) is shown under the blue/red column.

**Figure 4:**
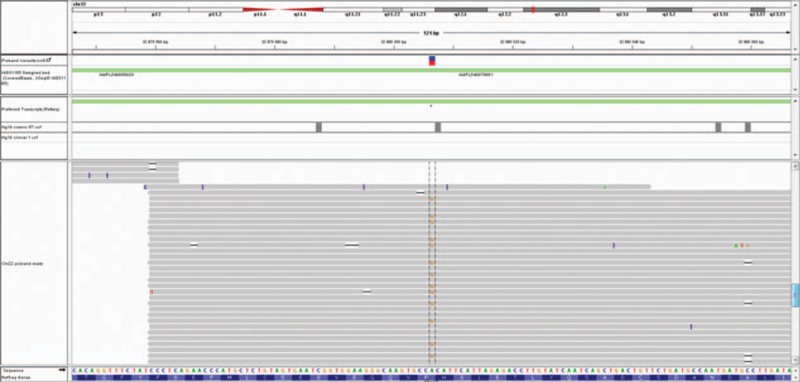
Visualization of F-box only protein 7 gene variant (NM_012179.3:c.540A > G,NP_001028196.1:p.Pro101=, rs41311141) in integrative genomics viewer. The variant G is shown under the blue/red column.

Molecular genetic examinations were performed due to the family history; a coding sequences, exon/intron regions and 5′/3′ untranslated region sequences of 16 genes (alcohol-dehydrogenase 1C gene, ATPase 13A3 gene, eukaryotic translation initiation factor 4 gamma 1 gene*, FBXO7* [F-box only protein 7 gene]*, GBA* [glucosylceramidase beta gene] + *GBAP1, GIGYF2* [GRB 10 interacting GYF protein 2], HtrA serine peptidase 2 gene, leucine-rich repeat kinase 2 gene*, MAPT* [microtubule associated protein tau gene], Parkinson disease protein 2 or parkin RBR E3 ubiquitin protein ligase gene, Parkinson disease protein 7, also known as DJ-1, PTEN induced putative kinase 1 gene, phospholipase A2 group VI gene*, SNCA* [synuclein alpha gene], ubiquitin C-terminal hydrolase L1 gene, and *VPS35* [vacuolar protein sorting 35 gene]) known to be associated with monogenic familial Parkinson disease was tested with a massive parallel sequencing method using Ion Torrent technology and confirmed by Sanger sequencing. In total, it covered 93% of gene sequences. The most common large copy number variants (CNV) known to be associated with Parkinson disease—*SNCA* gene duplication was tested by CNV comparison (NextGene 2.41—Softgenetics, PA, USA) with normal finding. No previously described causal mutation was found. After filtering against common variants (MAF < 0.01), 2 noncoding and 1 synonymous rare mutation potentially associable with parkinsonism were identified: *GIGYF2*, *PARK11* (c.∗2030G > A, rs115669549); *VPS35* gene—vacuolar protein sorting 35, *PARK17* (c.102 + 33G > A, rs192115886); and *FBXO7*, *PARK15* (c.540A > G, NP_001028196.1:p.Pro101=, rs41311141) (Figs. 3 and 4). The *VPS35* gene mutation was also confirmed in the patient's cousin and her 2 daughters. His cousin has mild late-onset Parkinson disease with the usual phenotype; her daughters have no symptoms of parkinsonism. All steps of the study were approved by the local ethics committee of the University Hospital Olomouc.

## 3 Discussion

Clinical pictures of the individual members in the researched pedigrees are different.^[1–3]^ In 2 cases, there is a typical, bilateral, symmetric, and L-DOPA responsive parkinsonism. These patients actually very well benefit from treatment with deep brain stimulation or continuous intestinal infusion of Duodopa^®^, Halden, Norway. In other cases, mostly in individuals older than 60 years, various combinations of bradykinesia, hypokinesia, rigidity, and tremor are present. These have varying severity, with varying responsiveness to dopaminergic therapy and are accompanied by varying degree of cognitive dysfunction. Considering that parkinsonian symptoms in these individuals are mostly asymmetric and only partially respond to L-DOPA therapy and given the type of cognitive deficit and the speed of its development (in relation to the beginning and duration of the disease), it seems that the morphological basis of this familial parkinsonism could be rather tauopathy than synucleinopathy. It could even be a combination of several neuropathological entities. Similarly, from the genetic point of view, more genetic abnormalities will probably play important role here. In our case, visual and auditory hallucinations have been reported, which in combination with cognitive deficit and parkinsonism could lead to a diagnosis of dementia with Lewy bodies (DLB). However, the hallucinations were only temporary and disappeared after antipsychotic treatment, and they did not appear again after its discontinuation. Furthermore, fluctuations of cognitive deficit were not present, another feature characteristic for DLB. Similarly, neither hallucinations nor fluctuating cognitive disorder were reported in any of the probands from researched pedigrees. Nevertheless, the youngest symptomatic subjects in pedigrees are aged 45 to 50 years.^[3]^ They suffer from only mild parkinsonism, and their cognitive functions are still normal; therefore, it is currently difficult to classify their disease and predict its next development.

The current case report presents a new phenotype variant of familial atypical parkinsonism, possibly associated with rare mutations in the *FBXO7* and *VPS35* genes. The *GIGYF2* “A” variant is most probably a neutral variant, as it is common in primates; the proposed association of any of the mutations with parkinsonism has not been confirmed.^[4]^ The positions in which the rare mutations in *VPS35* and *FBXO7* were described are phylogenetically conserved in primates. The *VPS35* (c.102 + 33G > A, rs192115886) variant was checked by NetGene2 software for the presence of donor/acceptor splice site around the variant, and in the case of wild-type “C” allele there were predicted 2 acceptor splice sites in direct strand while in the presence of “T” allele 1 splice site was silenced so the rare variant could result in alternative messenger RNA or messenger ribonucleic acid.

To evaluate the contribution of exonic synonymous variant in *FBXO7* gene (c.540A > G, rs41311141) in exonic isoform alteration we used Alternative Splice Site Predictor algorithm and there was predicted the exchange of cryptic donor site for cryptic acceptor site in the presence of the variant.

So it could be supposed that both rare variants (*VPS35* and *FBXO7)* can influence splicing.

The patient's disorder was diagnosed as “atypical parkinsonism sharing a progressive supranuclear palsy (PSP)—parkinsonism and Richardson syndrome phenotypes signs”. PSP is a rare neurodegenerative disease, usually presenting with predominant parkinsonism. However, 7 different phenotypes have been described.^[5]^ The combination of Parkinson syndrome with resting and postural tremor, early dysarthria, and dysphagia, together with progressive dementia, frequent falls, and supranuclear gaze palsy with apraxia of lid opening is not quite typical for any of these phenotypes. Although the familial form of PSP was not reported for many years after the first description, its existence has been repeatedly described in the last decade. In some cases, the presence of a causal mutation of *MAPT* or another gene was confirmed in these families.^[6–15]^ The *VPS35* gene at the *PARK17* locus encodes a key component of the retromer complex. It has been recently identified as a new cause of AD late-onset “sporadic” Parkinson disease with reduced penetrance.^[16–18]^*FBXO7* is a member of Skp1-Cullin-F-box-type E3 ubiquitin ligases, which play a substantial role in targeting proteins for ubiquitination. It is associated with autosomal-recessive and levodopa-responsive parkinsonism—pyramidal disease (PPD). PPD differs from Parkinson disease mainly in the juvenile onset and presence of limb spasticity. Four mutations have been identified in reported families with PPD.^[19–22]^

In contrast to previously reported cases and families carrying *VPS35* mutations, our patient had parkinsonism resembling PSP. He did not manifest any typical signs of PPD, such as juvenile onset or presence of pyramidal signs and spasticity; also, the course of his disease was typical for PSP tauopathy. Nevertheless, beside the typical PPD phenotype, we documented many more signs reported in the *FBXO7* in our patient—supranuclear gaze palsy, blepharospasm, eyelid apraxia, dysarthria, dysphagia, hypophonia, respiratory signs, and cognitive decline.^[20,23,24]^ From this point of view, this patient's disease would be more probably associated with the presence of the FBXO7 mutation of than with the VPS35 mutation.

As to the changes in the *FBXO7* and *VPS35* genes (despite phylogenetic conservation in primates), probably neither the *FBXO7* nor the *VPS35* variants will be direct causal mutations. Both described variants, and possibly the influence of their combination, could increase the risk of the disease.

## Supplementary Material

Supplemental Digital Content
